# 76. Idiopathic retroperitoneal fibrosis (possibly Ig G4 related)

**DOI:** 10.1093/rap/rky034.039

**Published:** 2018-09-20

**Authors:** May Zun Swe, Ameen Jubber, Rachel Jeffery, Mudita Tripathi

**Affiliations:** Rheumatology, Northampton General Hospital, Northampton, UNITED KINGDOM

**Introduction:** Retroperitoneal fibrosis is a rare condition characterised by the presence of inflammatory and fibrous retroperitoneal tissue that often encases the ureters or abdominal organs. It is a systemic autoimmune disease that may arise as a primary aortitis that elicits a periaortic fibroinflammatory response. It may be idiopathic (immune-mediated) or secondary to other causes such as drugs (ergot- derivatives, methysergide, bromocriptine, beta blockers, methyldopa), biological agents (etanercept, TNF-alpha blocker, infliximab), infections (TB, histoplasmosis, actinomycosis), malignancy, radiation therapy and surgery. Idiopathic forms account for over 70% of cases and are either IgG4 or non IgG4 related.

**Case description:** A 58 year old lady with chronic back pain secondary to a prolapsed intervertebral disc, migraine and depression, presented to A&E with a one-week history of worsening stabbing epigastric, left flank and groin pain, radiating to the back, associated with urinary retention, hesitancy and incomplete emptying of her bowels. She also complained of oral and genital ulcers as well as night sweats, nausea, fatigue, weight and appetite loss. She reported early morning stiffness affecting her shoulders, hands, pelvis and hips bilaterally. She has had a previous hysterectomy due to uterine cancer and a family history of lung and CNS malignancy was noted. She denied any recent travel history, contact with TB patients, and any exposure to asbestos. She was not sexually active and was not on any ergot derivatives for migraine. Examination findings were unremarkable apart from a tender epigastrium, oral and genital ulcerations. She has recently been investigated by gastroenterology and vascular team for longstanding back and abdominal symptoms that were experienced for the last 2-3 years. She was found to have an infra-renal abdominal aortic aneurysm measuring 3.8 cm with severe retroperitoneal fibrosis but no ureteric obstruction according to the CT abdomen and pelvis (2017). Subsequently, multiple investigations including autoimmune screen, infection screen and tumour markers were carried out to exclude secondary causes of retroperitoneal fibrosis. Both gastroscopy and colonoscopy including biopsy were not suggestive of cancer. CT angiogram aorta was performed following the recent admission, which showed a 3.8 cm abdominal aortic aneurysm, peri aortitis, moderate size hydro-nephrosis and right upper hydro-ureter. She was then started on prednisolone 1mg/ kg with gastric and bone protection as well as acyclovir prophylaxis.

**Discussion:** Although the diagnosis is often made by imaging studies, including CT scan or MRI, a definitive diagnosis may require a biopsy. However, many clinicians do not perform a biopsy in patients with imaging studies demonstrating findings typical of retroperitoneal fibrosis unless the patient is having surgery. The role of glucocorticoids is of paramount importance in treating retroperitoneal fibrosis and the extent of the disease can be monitored with a routine PET scan. Notably, urgent clinical advice should be sought from the renal team for ureteric stent insertion if hydronephrosis is complicated by obstructive uropathy.

**Key Learning Points:** Firstly, the initial clinical features of idiopathic or secondary retroperitoneal fibrosis are nonspecific, and the diagnosis is often not considered until there is significant organ (most commonly kidney) involvement. Most patients have ureteral obstruction and renal impairment by the time they come to medical attention. In addition, many patients have anaemia, possibly related to renal insufficiency or chronic inflammation. Furthermore, among patients with presumed idiopathic retroperitoneal fibrosis, ANA, IgG4, ASMA, ANCA, TFTs, thyroid microsomal antibody and thyroglobulin are normally checked. ANA has been reported to be positive in up to 50% of cases and antibodies against thyroid microsome and thyroglobulin are positive in approximately 25% of cases.

**Disclosure: M. Swe:** None. **A. Jubber:** None. **R. Jeffery:** None. **M. Tripathi:** None.

